# Septicemia is associated with increased risk for dementia: a population-based longitudinal study

**DOI:** 10.18632/oncotarget.20899

**Published:** 2017-09-15

**Authors:** Chung-Hsing Chou, Jiunn-Tay Lee, Chun-Chieh Lin, Yueh-Feng Sung, Che-Chen Lin, Chih-Hsin Muo, Fu-Chi Yang, Chi-Pang Wen, I-Kuan Wang, Chia-Hung Kao, Chung Y. Hsu, Chun-Hung Tseng

**Affiliations:** ^1^ Department of Neurology, Tri-Service General Hospital, National Defense Medical Center, Taipei, Taiwan, Republic of China; ^2^ Graduate Institute of Medical Sciences, National Defense Medical Center, Taipei, Taiwan, Republic of China; ^3^ Management Office for Health Data, China Medical University Hospital, Taichung, Taiwan, Republic of China; ^4^ Institute of Population Health Sciences, National Health Research Institutes, Zhunan, Taiwan, Republic of China; ^5^ Graduate Institute of Clinical Medical Science, China Medical University, Taichung, Taiwan, Republic of China; ^6^ Department of Internal Medicine, College of Medicine, China Medical University, Taichung, Taiwan, Republic of China; ^7^ Division of Kidney Disease, China Medical University Hospital, Taichung, Taiwan, Republic of China; ^8^ Department of Nuclear Medicine, PET Center, China Medical University Hospital, Taichung, Taiwan, Republic of China; ^9^ Department of Neurology, China Medical University Hospital, Taichung, Taiwan, Republic of China

**Keywords:** septicemia, infection, dementia, Alzheimer's disease, non-Alzheimer dementias

## Abstract

**Background:**

Systemic infection has been linked to cognitive impairment. We hypothesized that patients with septicemia are predisposed to increased risks for developing dementia in a long-term setting.

**Methods:**

This observational, retrospective, longitudinal, nation-wide population-based study was conducted using the data deduced from Longitudinal Health Insurance Database (LHID) in Taiwan. All patients with septicemia hospitalized for the first time from 2001 to 2011 without prior dementia were included. The development of Alzheimer's disease (AD) or non-Alzheimer dementias (NAD) in relation to the development of septicemia for each patient was recorded. An age- and sex-matched cohort without septicemia and without prior dementia served as the control. Septicemia, dementia, and other confounding factors were defined according to International Classification of Diseases Clinical Modification Codes. Cox proportional-hazards regressions were utilized to analyze adjusted hazard ratios.

**Results:**

Patients with septicemia had a higher risk for developing dementia based on hazard ratios (HRs) (*p*<0.001). Patients with septicemia in the younger age groups had a greater dementia risk (*p*<0.01). Septicemia was associated with subsequent NAD (*p*<0.001), whereas the increased risk of AD was statistically insignificant (*p*>0.05). Furthermore, higher severity of septicemia was associated with increased risk of developing dementia.

**Conclusions:**

Our findings suggest that septicemia is associated with an increased risk in developing NAD but not AD. A likely causal role of septicemia in increasing the risk of NAD is suggested, according to the findings that patients with higher severity of septicemia carried greater risk of sustaining dementia.

## INTRODUCTION

Septicemia is a serious and even life-threatening medical condition which is characterized by the presence of bacteria or bacterial toxin in blood circulation. It is accompanied by a systemic inflammatory response following initial attempts to eliminate pathogens. The aberrant responses are shown to deteriorate the permeability of the blood-brain barrier, and possibly compromise cognitive functions [1, 2]. A recent study reported that patients surviving severe sepsis suffer from long-term cognitive impairment in association with significant disability or higher mortality [3]. Sepsis, the result of septicemia, has been demonstrated to have a link to brain dysfunctions and degeneration. However, the role of septicemia in the development of dementia remains sketchy in a long-term setting.

Dementia is one of the major causes of disability. A relative increase in the aging population worldwide has made dementia a serious issue for individuals, family and society. Dementia is a degenerative brain disorder, which increases with age in general population. It has been suggested to be associated with many co-morbidities including stroke, diabetes mellitus, hyperlipidemia and hypertension [4, 5]. Systemic inflammatory response has been suggested to increase the brain's susceptibility to neurodegenerative disease, further deterioration of cognitive ability, and risk of developing dementia in later life [1, 6, 7]. A recent study has shown that patients with sepsis were likely to develop dementia in comparison with control group, whereas no specific dementia was pointed to [8]. It is of interest to determine whether septicemia affects selected types of dementia.

We hypothesized that septicemia, a potential cause of sepsis, increases risk of dementia. We used the database from NHI program which represents 99% of the population in Taiwan, to explore whether a history of septicemia is associated with subsequent increase in the risk of developing Alzheimer's disease (AD) or non-Alzheimer dementias (NAD).

## RESULTS

A total of 20,466 patients with septicemia and 40,932 age- and sex-matched controls for comparison were included in this observational cohort study. The baseline demographic and clinical characteristics of septicemic and control subjects are presented in Table 1. No difference in distribution of age and sex is noted between groups. Incidences of all co-morbidities are significantly greater in the septicemia than the comparison cohort (*p*<0.0001 for every co-morbidity).

**Table 1:** Baseline demographic status and comorbidity between comparison and septicemia groups

Variable	Comparison cohortN = 40932 (%)	Septicemia cohortN = 20466 (%)	*p*-value
Age, years (SD)^*^	65.4 (16.7)	65.6 (16.8)	0.1266
<45	5568 (13.6)	2784 (13.6)	
45-64	11510 (28.1)	5755 (28.1)	
≥65	23854 (58.3)	11927 (58.3)	
Sex			>0.99
Female	17998 (44.0)	8999 (44.0)	
Male	22934 (56.0)	11467 (56.0)	
Co-morbidities			
Stroke	2539 (6.2)	4022 (19.7)	<0.0001
DM	6605 (16.1)	6483 (31.7)	<0.0001
Hyperlipidemia	12828 (31.3)	7061 (34.5)	<0.0001
Hypertension	21385 (52.2)	13255 (64.8)	<0.0001
Depression	3219 (7.86)	2472 (12.1)	<0.0001
ARD	1253 (3.06)	2005 (9.80)	<0.0001
Smoking	17939 (43.8)	11159 (54.5)	<0.0001
NSAID use	21997 (53.7)	8849 (43.2)	<0.0001

Abbreviation: ARD: alcoholism-related disease; DM: diabetes mellitus; NSAID: non-steroidal anti-inflammatory drug; SD: standard deviation.

^*^t-test.

**Figure 1:**
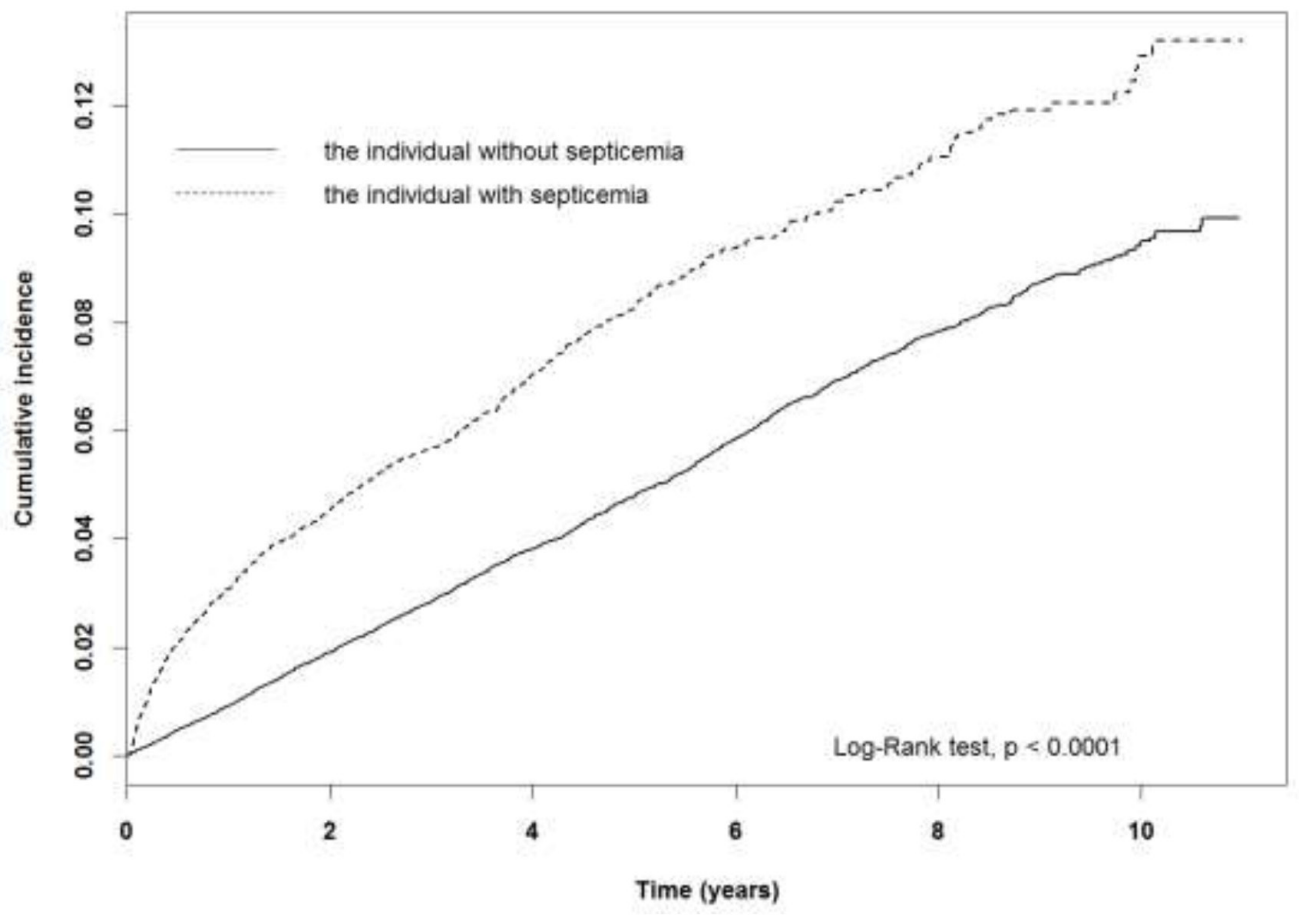
The dementia cumulative incidence curves for the individual with and without septicemia

As shown in Table 2, the overall incidences of dementia were approximately 2.14-fold higher in septicemia (95% CI: 1.97–2.34, *p*<0.001) than in the comparison cohort. The dementia incidence rate in the septicemia and comparison cohorts were 183 and 99.4 per 10,000 person-years respectively. The cumulative incidence curve revealed a significantly higher incidence in the septicemia than the comparison cohort (Figure 1) (log rank test *p*<0.0001). In addition, adjusted to subtypes of dementia, the HR of NAD (2.26, 95% CI: 2.07–2.47, *p*<0.001) is significantly higher in the septicemia than the comparison cohort. However, no significant difference in HRs for AD is noted between groups (HR = 1.17, 95% CI: 0.84–1.62, *p*>0.05).

**Table 2:** Incidence of dementia and dementia subtype and multivariate Cox proportional hazards regression analysis measured hazard ratios for study cohort

Variable	Comparison cohort			Septicemia cohort			Crude HR (95% CI)	Adjusted HR (95% CI)
	Event	PYs	Rate	Event	PYs	Rate		
All dementia	1945	195610	99.4	832	45425	183	1.79 (1.65-1.94)^***^	2.09 (1.92-2.28)^***^
Alzheimer's disease	222	195610	11.4	46	45425	10.1	0.89 (0.64-1.22)	1.15 (0.83-1.60)
non-Alzheimer dementias	1723	195610	88.1	786	45425	173	1.91 (1.75-2.07)^***^	2.20 (2.01-2.41)^***^

Model adjusted for age, sex, stroke, DM, hyperlipidemia, hypertension, depression, ARD, smoking, and NSAID use.

Abbreviation: PYs: person-years; Rate: incidence rate, per 10,000 person-years; ARD: alcoholism-related disease; DM: diabetes mellitus; NSAID: non-steroidal anti-inflammatory drug.

^***^p<0.001.

We note an increasing trend for developing dementia with increasing severity of septicemia based on hospitalization length (*p* for trend <0.0001) using multivariate Cox proportional hazards regression analysis (Table 3). Results show that, adjusted for age, sex and co-morbidities, HRs for dementia risk were 1.97 (95% CI: 1.66–2.35, *p*<0.001), and 2.18 (95% CI: 1.99–2.39, *p*<0.001) for the length of septicemia hospitalization 1–5 days and ≥ 5 days, respectively. There were the same trend for the association between the life time portion of septicemic hospitalization and dementia. Compared to comparisons, the highest risk were 1.23 (95% CI = 1.08–1.40, *p*<0.01), 3.47 (95% CI = 3.10–3.86, *p*<0.001), and 5.08 (95% CI = 4.02–6.43, *p*<0.001) for mild, moderate and severe septicemia.

**Table 3:** Incidence of dementia and multivariate Cox proportional hazards regression analysis measured hazard ratios for study cohort by severity of septicemia

Severity	N	Event	PYs	Rate	Crude HR (95% CI)	Adjusted HR (95% CI)
Length of stay due to septicemia						
Comparisons	40932	1945	195610	99.4	ref	ref
1-5 days	4733	135	11318	119.3	1.19 (1.00-1.42)^***^	1.92 (1.61-2.29)^***^
> 5 days	15733	697	34107	204.4	1.99 (1.82-2.17)^***^	2.13 (1.94-2.33)^***^
p for trend					<0.0001	<0.0001
Severity of septicemia						
Comparisons	40932	1945	195610	99.4	ref	ref
Mild (T1)	6306	274	32718	83.8	0.84 (0.74-0.96)^**^	1.20 (1.06-1.37)^**^
Moderate (T2)	6640	472	11719	403	3.96 (3.57-4.39)^***^	3.37 (3.02-3.76)^***^
Severe (T3)	7520	86	988	871	7.74 (6.13-9.78)^***^	5.04 (3.98-6.37)^***^
p for trend					<0.0001	<0.0001

Model adjusted for age, sex, stroke, DM, hyperlipidemia, hypertension, depression, ARD, smoking, and NSAID use.

Abbreviation: PYs: person-years; Rate: incidence rate, per 10,000 person-years; CI, confidence interval; ARD: alcoholism-related disease; DM: diabetes mellitus; NSAID: non-steroidal anti-inflammatory drug; HR, hazard ratio; ref: reference group; T, tertile.

^**^p<0.01; ^***^p<0.001.

Severity = (total length of hospital stay due to septicemia during the follow-up duration) ÷ (length of follow-up duration).

T1, the first tertile:<1.5%; T2, the second tertile: 1.5-50%; T3 the third tertile:>50%.

Table 4 demonstrated the risk of dementia between the individual with and without septicemia stratified by demographic factor and co-morbidities. Results show that the individuals with septicemia were likely to develop all-cause dementia compared to those without septicemia stratified by age, sex and co-morbidities. Notably, patients with NAD, but not AD, contributed to the difference in dementia incidence rate and accordingly HRs between septicemia and comparison cohorts. Patients aged <45 yrs exhibited the highest risk of developing all-cause dementia (HR = 8.54, 95% CI: 2.25–32.4, *p*<0.01), followed by patients aged 45 to 64 yrs (HR = 3.20, 95% CI: 2.33–4.42, *p*<0.001) and those aged ≥65 yrs (HR = 1.85, 95% CI: 1.69–2.02, *p*<0.001). Additionally, the difference in HR of developing AD or NAD between females and males was statistically insignificant.

**Table 4:** Demographic factors and comorbidities stratified analysis estimated hazard ratios of dementia risk in the individual with and without septicemia

Variable	Comparison cohort			Septicemia cohort			Adjusted HR (95% CI)
	Event	PYs	Rate	Event	PYs	Rate	
All dementia							
Age group							
<45	3	30347	0.99	8	10733	7.45	7.32 (1.85-28.9)^**^
45-64	80	60181	13.3	94	15692	59.9	2.97 (2.15-4.11)^***^
≧65	1862	105082	177	730	18999	384	1.80 (1.65-1.97)^***^
Sex							
Female	974	86350	113	428	22742	188	1.94 (1.72-2.19)^***^
Male	971	109260	88.9	404	22683	178	2.26 (2.00-2.56)^***^
Co-morbidities							
No	305	83709	36.4	92	15263	60.3	3.32 (1.94-5.68)^***^
Yes	1640	111901	144	740	30161	245	2.32 (2.13-2.52)^***^
Alzheimer's disease							
Age group							
<45	0	30347	0.00	0	10733	0.00	NA
45-64	10	60181	1.66	4	15692	2.55	0.97 (0.28-3.30)
≧65	212	105082	20.2	42	18999	22.1	1.07 (0.76-1.51)
Sex							
Female	137	86350	15.9	31	22742	13.6	1.16 (0.77-1.73)
Male	85	109260	7.78	15	22683	6.61	1.15 (0.65-2.03)
Co-morbidities							
No	47	83709	5.61	5	15263	3.28	1.16 (0.15-8.76)
Yes	175	111901	15.6	41	30161	13.6	1.13 (0.82-1.57)
non-Alzheimer dementias							
Age group							
<45	3	30347	0.99	8	10733	7.45	7.32 (1.85-28.9)^**^
45-64	70	60181	11.6	90	15692	57.4	3.27 (2.33-4.60)^***^
≧65	1650	105082	157	688	18999	362	1.89 (1.72-2.07)^***^
Sex							
Female	837	86350	96.9	397	22742	175	2.06 (1.82-2.34)^***^
Male	886	109260	81.1	389	22683	171	2.36 (2.08-2.68)^***^
Co-morbidities							
No	258	83709	30.8	87	15263	57.0	3.80 (2.17-6.66)^***^
Yes	1465	111901	131	699	30161	232	2.47 (2.27-2.70)^***^

Model adjusted for age, sex, stroke, DM, hyperlipidemia, hypertension, depression, ARD, smoking, and NSAID use.

Abbreviation: PYs: person-years; Rate: incidence rate, per 10,000 person-years; CI, confidence interval; ARD: alcoholism-related disease; DM: diabetes mellitus; NSAID: non-steroidal anti-inflammatory drug; HR: hazard ratio.

^**^p<0.01; ^***^p<0.001.

The propensity score matching method was applied to the study and comparison cohorts with 1:1 ratio, and the results were shown in Supplementary Tables 1-4. In general, all the significant adjusted HRs remain using the propensity score matching method, except adjusted HR for Mild (T1) severity in Table 3, Age group <45 and No co-morbidities in NAD groups in Table 4, with a higher *p* value, due to a relatively small event number.

## DISCUSSION

This population-based longitudinal study was performed to assess whether septicemia, an acute systemic infection, was associated with an increased risk for developing dementia. We found that patients with septicemia had a significantly higher risk for developing dementia. It is noted that young patients with septicemia were at significantly higher risk of developing dementia. The incidence of all-cause dementia among the group of young age (<45 for example) in the comparison cohort is lower than those in the groups of elder age. The cases of young patients with septicemia who developed subsequent dementia therefore significantly increased the incidence and the odds ratio of developing dementia in the young age group. Furthermore, in the age group of <45, patients with NAD rather than AD (event 0) contributes to the increased risk of developing all-cause dementia in patients with septicemia. Also, it is likely that increasing age introduce additional causes for developing NAD, resulting in the dilution of the impact of septicemia on NAD risk.

In the present study, it is interesting to note that septicemia is associated with an increased risk of subsequent NAD, but not AD. Currently, the evidence compiled from the literature linking AD to an infectious cause is inconclusive although the amount of evidence suggestive of an association is too substantial to ignore [9]. The risks of specific types of dementia may be differentially affected in septicemia patients with different pathogens. *Borrelia burgdorferi*, a type of pathogens causing Lyme disease, for example, has been evaluated extensively for a possible etiologic link to AD. A pooled analysis of studies investigating the association between *B. burgdorferi* and AD found that *B. burgdorferi* was 13 times more frequent in the brains of patients with AD than in brains of controls [10]. A relatively low prevalence of causative pathogen of AD, such as *B. burgdorferi* may account for a non-significant relationship between AD and septicemia in Taiwan [11].

Dementia has been suggested to increase with age, and most studies focused on subjects aged over 65. In the present study, patients with septicemia were demonstrated to be at a greater risk for developing dementia in all age groups, including relatively young subjects aged 20–44 and 45–64 years. It suggests that septicemia contributes to dementia patients aged less than 65 years, known as early onset dementia. It has been suggested that for septicemia patients who had subsequent stroke, regardless of the onset age, are at a high risk of developing poststroke dementia resulting from a complex interplay between stroke lesion features and brain resilience [12]. Correlation of septicemia with early onset dementia, however, requires further controlled studies to elucidate. It has been revealed that the prevalence of dementia in people aged 65 and over was 5.7% in Taiwan [4]. Nevertheless, for people aged 65 and over in our comparison and septicemia cohorts, the incidence of dementia was 1.77% and 3.84% respectively. The diagnosis of dementia in our study population was conferred by various diagnosing tools. It suggests that their disease state may be more severe than other studies in which patients were evaluated by primary screening instruments for dementia.

Septicemia is considered as an independent factor accounting for the increased risk of developing dementia. It is acknowledged that dementia is a multifactorial disorder and contribution of co-morbidities in study groups may stand. In this study, the prevalence of all the co-morbidities included in the septicemia cohort was significantly higher than the comparison cohort. The increased risk of dementia in the septicemic patients was observed in both groups with or without each specific type of co-morbidities, including stroke, diabetes mellitus, hyperlipidemia and hypertension. In consistent with a recent population-based study which suggested that dementia was associated with prior sepsis, regardless of sex [8], we found an increased risk of dementia in the septicemia cohort in both sex groups.

In the present study, patients with a higher severity of septicemia have a greater risk for developing dementia. This finding is a support to a previous case-control study in the UK, which demonstrated a positive association between episodes of infection in the four years preceding diagnosis of dementia and increased likelihood of diagnosis of dementia [13]. The odds ratio of developing dementia in age group of 85 and over who had two or more infective episodes was 1.4, compared to those who had none or single episode [13].

Septicemia, a potential cause of sepsis resulting in intense inflammation response, may lead to extensive brain degeneration [14]. Our findings that septicemia as a risk factor for developing NAD is postulated to be attributed to the sepsis-induced neuroinflammation [14]. Vascular endothelium has been shown to be affected by immune activation induced by lipopolysaccharide (LPS) or invasion directly by virulent bacteria in the bloodstream during sepsis [15–17]. Cognitive impairment, as a consequence of septicemic assaults, may result from damage of vascular vessels or the blood-brain barrier (BBB) [14]. Vascular dementia, belonging to NAD, is also associated with hemodynamic disturbance as well as embolic stroke during episodes of septicemia [18]. In our previous study, there was an increased risk of stroke after septicemia, based on a population-based longitudinal database in Taiwan [19]. This is in line with the increased risk of cerebral vascular damage in patients with septicemia, who developed post-stroke dementia, as a consequence [20, 21]. In contrast to AD and some other forms of dementia, onset and acceleration of cognitive impairment due to sepsis is likely partially preventable in many patients [6, 9]. The high economic burden, especially for young patients who developed early onset dementia after septicemia, can therefore be lessened [22].

Etiological pathogens have been identified in the circulating blood stream in the septicemia cohort of the present study, and it indicates synergistic effects of direct invasion by pathogens and indirect influence of toxins and immune activation to the central nervous system [23]. Specific pathogens, such as varicella zoster virus, influenza, Helicobacter pylori (Hp), Chlamydia pneumoniae, as well as periodontitis have been reported to be associated with stroke and potentially subsequent vascular dementia, a subtype of NAD [24, 25]. A positive correlation between Hp infection and carotid atherosclerosis, for instance, has been demonstrated in patients with vascular dementia [26]. Moreover, the prospective Northern Manhattan Study suggests that atherosclerosis is associated with infectious burden index, serologically measured for five pathogens, including Hp, Chlamydia pneumonia, cytomegalovirus, and herpes simplex virus 1 and 2 [27, 28]. The interest of the present study is the overall systemic response to various pathogens, which make patients predisposed to subsequent dementia, and a positive correlation between severity of septicemia and increased risks for developing NAD, not AD, in a long-term setting was demonstrated. Future studies are required to investigate the underlying mechanisms, such as atherosclerosis, by which septicemia makes patients predisposed to the development of NAD.

There are several limitations to this study. First of all, the database provides no lifestyle or health information such as intensity of tobacco use, alcohol consumption, exercise habits and diet that may influence the risk of AD, NAD and stroke. Secondly, data from the dataset may also include unidentified recurrent patients who may have had septicemia and stroke before 1996 when NHI was started [29]. Nevertheless, we only included patients with new incidences of dementia and septicemia in order to increase the accuracy of the diagnosis of dementia and septicemia. Although our study depended on ICD codes, which may be different from other studies, the NHI administration in Taiwan has a cross-checking system to evaluate the precision of records from different hospitals [30].

## MATERIALS AND METHODS

### Data source

This observational study was conducted using the data from the Longitudinal Health Insurance Database (LHID), which is a sub-database from the Taiwan National Health Insurance program (Taiwan NHI) covering over 99% of 23 million Taiwan citizens. LHID contains data from original claims of one million beneficiaries, randomly sampled from the registry for the Beneficiaries of the National Health Insurance Research Database (NHIRD) from the National Health Insurance (NHI) program, the universal payer for healthcare in Taiwan [31]. The database included the inpatient and outpatient information of disease history of all insured people based on International Classification of Diseases, Ninth Revision, Clinical Modification (ICD-9-CM). Taiwanese government gave a scrambled and anonymous number before releasing data for research to safeguard the privacy of the insured people. NHI randomly audits the healthcare records to validate medical claims for diagnosis and treatment. A disease diagnosis without valid supporting clinical findings may be considered a medical fraud by NHI with a penalty of 100-fold of the payment claimed by the treating physician or hospital. Previous reports have shown the reliability of the diagnosis coding in the LHIC [32, 33]. This study protocol was reviewed and approved by the Ethics Review Board of China Medical University (CMUH104-REC2-115).

### Study population

This study was a retrospective population-based cohort study. A septicemia cohort and a comparison cohort without septicemia were established for assessing potential difference in the risk of developing dementia between groups. The septicemia cohort contains patients carrying a diagnosis of septicemia (ICD-9-CM 003.1, 036.2 and 038, in hospital) from 2001 to 2011. The index date of septicemia cohort was the first date of septicemia diagnosis. The comparison cohort contains individuals without diagnosis of septicemia in the same LHID, matched by age and sex at 1:2 ratios. We excluded any individual with a prior diagnosis of dementia, followed these study subjects and terminated the follow-up when the individual withdrew from NHI, developed dementia or until December 31, 2011. The primary outcome was the diagnosis of dementia (ICD-9-CM 290, 294.1 and 331.0). We also investigated the risk of subtype of dementia: Alzheimer's disease (ICD-9-CM 331.0) and non-Alzheimer dementias (ICD-9-CM 290.0–290.4).

The confounding factors included in the present study are age, sex and co-morbidities that are relevant to dementia. Comorbidities included stroke (ICD-9-CM 430–437), diabetes mellitus (DM, ICD-9-CM 250), hyperlipidemia (ICD-9-CM 272), hypertension (ICD-9-CM 401–405), depression (ICD-9-CM 296.2-296.3, 300.4 and 311), alcoholism-related disease (ARD, ICD-9-CM 291, 303, 305, 571.0, 571.1, 571.2, 571.3, 790.3 and V11.3), smoking (ICD-9-CM 305.1, 649.0 and 490-496), and use of nonsteroidal anti-inflammatory drugs (NSAID, ATC code M01A).

### Statistical analysis

We presented the age distribution as mean and standard deviation (SD) and sex and co-morbidities distribution as number and percentage between septicemia and comparison cohorts and compared the distribution difference by t-test (for age) and chi-square test (for sex and co-morbidities). The incidence density of dementia was compared between groups with the total number of dementia diagnosis divided by the total sum of follow-up years (per 10,000 person-years). We measured and compared the cumulative incidence curves between septicemia and comparison cohorts by Kaplan-Meier method and tested the difference between groups by log-rank test. The hazard ratios (HRs) and 95% confidence intervals (CIs) for dementia risk between septicemia and comparison cohorts were analyzed using single-variable and multi-variable Cox proportional hazard models. Association between the severity of septicemia and dementia was also estimated. The septicemic severity was defined based on two methods: 1. the length of stay at the first admission of septicemia, and 2. the life time portion of septicemic hospitalization: the length of stay due to septicemia during the study period divided by the total length of follow-up time [34–37]. The risks of dementia in the cohorts with and without septicemia were calculated with stratification by age, sex and co-morbidities. The propensity score was calculated using a logistic regression model that contained the variables of age, sex, stroke, DM, hyperlipidemia, hypertension, depression, ARD, smoking, and NSAID use. The c-statistic was 0.853. We managed and analyzed the data by SAS 9.4 software (SAS Institute, Cary, NC, USA) and draw the incidence curve by R software (R Foundation for Statistical computing, Vienna, Austria). A two-tailed *p* value less than 0.05 was considered statistically significant.

## CONCLUSION

In conclusion, septicemia was linked to an increased risk of developing NAD, but not AD. Young patients were more vulnerable to develop dementia after surviving septicemia. Patients who had experienced higher severity of septicemia had a greater risk of developing dementia. These findings suggest that preventive measures of dementia could be improved by preventing and treating septicemia, especially for young people.

## SUPPLEMENTARY MATERIALS TABLES



## Abbreviations

LHID: Longitudinal Health Insurance Database
AD: Alzheimer's disease
NAD: non-Alzheimer dementias
ICD: International Classification of Diseases
PYs: person-years
CI: confidence interval
DM: diabetes mellitus
HR: hazard ratio
ref: reference group
T: tertile
DM: diabetes mellitus
SD: standard deviation
