# Supplementary material for: Septicemia is associated with increased risk for dementia: a population-based longitudinal study
Source: Oncotarget. 2017 Sep 15;8(48):84300–8. doi: 10.18632/oncotarget.20899 (PMC5663596; doi:10.18632/oncotarget.20899)
Supplement: Supplementary file 1 [file oncotarget-08-84300-s001.pdf]

Septicemia is associated with increased risk for dementia: a population-based longitudinal study

SUPPLEMENTARY MATERIALS

Supplementary Table 1: Baseline demographic status and comorbidity between comparison and septicemia groups

Variable	Comparison cohort N = 20406 (%)	Septicemia cohort N = 20406 (%)	p-value
Age, years (SD)*	65.4 (16.7)	65.5 (16.8)	0.53
<45	2744 (13.5)	2784 (13.6)	0.83
45-64	5786 (28.4)	5755 (28.2)	
≥65	11876 (58.2)	11867 (58.2)	
Sex			0.79
Female	8957 (43.9)	8984 (44.0)	
Male	11449 (56.1)	11422 (56.0)	
Co-morbidities			
Stroke	3916 (19.2)	3965 (19.4)	0.54
DM	6465 (31.7)	6444 (31.6)	0.82
Hyperlipidemia	7068 (34.6)	7051 (34.6)	0.86
Hypertension	13224 (64.8)	13196 (64.7)	0.77
Depression	1968 (9.64)	1948 (9.55)	0.74
ARD	1660 (8.13)	1607 (7.88)	0.33
Smoking	11090 (54.4)	11106 (54.4)	0.87
NSAID use	8988 (44.1)	8847 (43.4)	0.13

Abbreviation: ARD: alcoholism-related disease; DM: diabetes mellitus; NSAID: non-steroidal anti-inflammatory drug; SD: standard deviation.
*t-test.

Supplementary Table 2: Incidence of dementia and dementia subtype and multivariate Cox proportional hazards regression analysis measured hazard ratios for study cohort

Variable	Comparison cohort			Septicemia cohort			Crude HR (95% CI)	Adjusted HR (95% CI)
	Event	PYs	Rate	Event	PYs	Rate		
All dementia	925	87155	106.13	826	45384	182.00	1.64 (1.50-1.81)***	2.05 (1.86-2.26)***
Alzheimer's disease	86	87155	9.87	46	45384	10.14	1.02 (0.71-1.46)	1.23 (0.85-1.77)
non-Alzheimer dementias	839	87155	96.27	780	45384	171.87	1.71 (1.55-1.88)***	2.14 (1.93-2.36)***

Model adjusted for age, sex, stroke, DM, hyperlipidemia, hypertension, depression, ARD, smoking, and NSAID use.
Abbreviation: PYs: person-years; Rate: incidence rate, per 10,000 person-years; ARD: alcoholism-related disease; DM: diabetes mellitus; NSAID: non-steroidal anti-inflammatory drug.
***$p<0.001$.

Supplementary Table 3: Incidence of dementia and multivariate Cox proportional hazards regression analysis measured hazard ratios for study cohort by severity of septicemia

Severity	N	Event	PYs	Rate	Crude HR (95% CI)	Adjusted HR (95% CI)
Length of stay due to septicemia						
Comparisons	20406	925	87155	106.13	Ref.	Ref.
1-5 days	4722	135	11314	119.32	1.13 (0.94-1.35)***	1.94 (1.62-2.33)***
> 5 days	15684	691	34070	202.82	1.81 (1.64-2.00)***	2.08 (1.88-2.30)***
p for trend					<0.0001	<0.0001
Severity of septicemia						
Comparisons	20406	925	87155	106.13	Ref.	Ref.
Mild (T1)	6302	273	32709	83.46	0.80 (0.69-0.91)***	1.18 (1.03-1.36)*
Moderate (T2)	6617	469	11691	401.16	3.50 (3.12-3.93)***	3.16 (2.81-3.55)***
Severe (T3)	7487	84	984	853.62	6.11 (4.79-7.80)***	4.22 (3.31-5.38)***
p for trend					<0.0001	<0.0001

Model adjusted for age, sex, stroke, DM, hyperlipidemia, hypertension, depression, ARD, smoking and NSAID use.
Abbreviation: PYs: person-years; Rate: incidence rate, per 10,000 person-years; CI, confidence interval; ARD: alcoholism-related disease; DM: diabetes mellitus; NSAID: non-steroidal anti-inflammatory drug; HR, hazard ratio; ref: reference group; T, tertile.
*p<0.05; ***p<0.001.
Severity = (total length of hospital stay due to septicemia during the follow-up duration) ÷ (length of follow-up duration).
T1, the first tertile:<1.5%; T2, the second tertile: 1.5-50%; T3 the third tertile:>50%.

Supplementary Table 4: Demographic factors and comorbidities stratified analysis estimated hazard ratios of dementia risk in the individual with and without septicemia

Variable	Comparison cohort			Septicemia cohort			Adjusted HR (95% CI)
	Event	PYs	Rate	Event	PYs	Rate	
All dementia							
Age group							
<45	1	14736	0.68	8	10733	7.45	11.8 (1.47-95.5)*
45-64	54	48612	18.87	94	15692	59.90	3.19 (2.27-4.48)***
≧65	870	43807	198.60	724	18959	381.89	1.91 (1.72-2.11)***
Sex							
Female	448	39241	114.17	424	22723	186.59	1.93 (1.68-2.21)***
Male	477	47914	99.55	402	22661	177.40	2.18 (1.90-2.50)***
Co-morbidities							
No	29	7989	36.30	17	3694	46.02	2.03 (1.11-3.72)*
Yes	896	79166	113.18	809	41690	194.05	2.03 (1.84-2.23)***
Alzheimer's disease							
Age group							
<45	0	14736	0.00	0	10733	0.00	NA
45-64	4	28612	1.40	4	15692	2.55	1.54 (0.38-6.26)
≧65	82	43807	18.72	42	18959	22.15	1.18 (0.81-1.73)
Sex							
Female	47	39241	11.98	31	22723	13.64	1.37 (0.86-2.18)
Male	39	47914	8.14	15	22661	6.62	1.01 (0.55-1.87)
Co-morbidities							
No	3	7989	3.76	1	3694	2.71	1.09 (0.11-10.7)
Yes	83	79166	10.48	45	41690	10.79	1.25 (0.86-1.80)
Non-Alzheimer dementias							
Age group							
<45	1	14736	0.68	8	10733	7.45	11.8 (1.47-95.5)*
45-64	50	28612	17.48	90	15692	57.35	3.32 (2.34-4.72)***
≧65	788	43807	179.88	682	18959	359.73	1.98 (1.78-2.20)***
Sex							
Female	401	38241	102.19	393	22723	172.95	1.99 (1.73-2.30)***
Male	438	47914	91.41	387	22661	170.78	2.28 (1.98-2.63)***
Co-morbidities							
No	26	7989	32.55	16	3694	43.31	2.15 (1.14-4.03)*
Yes	813	79166	102.70	764	41690	183.26	2.11 (1.91-2.33)***

Model adjusted for age, sex, stroke, DM, hyperlipidemia, hypertension, depression, ARD, smoking, and NSAID use.
Abbreviation: PYs: person-years; Rate: incidence rate, per 10,000 person-years; CI, confidence interval; ARD: alcoholism-related disease; DM: diabetes mellitus; NSAID: non-steroidal anti-inflammatory drug; HR: hazard ratio.
*p <0.05; ***p<0.001.
